# Evaluation of ultrasound Tissue Velocity Imaging: a phantom study of velocity estimation in skeletal muscle low-level contractions

**DOI:** 10.1186/1471-2342-13-16

**Published:** 2013-06-07

**Authors:** Frida Lindberg, Mattias Mårtensson, Christer Grönlund, Lars-Åke Brodin

**Affiliations:** 1 School of Technology and Health, Royal Institute of Technology (KTH), Huddinge, Sweden; 2 Department of Biomedical Engineering – R&D, Radiation science, Umeå University, Umeå, Sweden

**Keywords:** Tissue Velocity Imaging, Ultrasound, Skeletal muscle, Phantom evaluation, Pulse repetition frequency

## Abstract

**Background:**

Tissue Velocity Imaging (TVI) is an ultrasound based technique used for quantitative analysis of the cardiac function and has earlier been evaluated according to myocardial velocities. Recent years several studies have reported applying TVI in the analysis of skeletal muscles. Skeletal tissue velocities can be very low. In particular, when performing isometric contractions or contractions of low force level the velocities may be much lower compared to the myocardial tissue velocities.

**Methods:**

In this study TVI was evaluated for estimation of tissue velocities below the typical myocardial velocities. An in-house phantom was used to see how different PRF-settings affected the accuracy of the velocity estimations.

**Results:**

With phantom peak velocity at 0.03 cm/s the error ranged from 31% up to 313% with the different PRF-settings in this study. For the peak velocities at 0.17 cm/s and 0.26 cm/s there was no difference in error with tested PFR settings, it is kept approximately around 20%.

**Conclusions:**

The results from the present study showed that the PRF setting did not seem to affect the accuracy of the velocity estimation at tissue velocities above 0.17 cm/s. However at lower velocities (0.03 cm/s) the setting was crucial for the accuracy. The PRF should therefore preferable be reduced when the method is applied in low-level muscle contraction.

## Background

Tissue Velocity Imaging (TVI) is an ultrasound based technique used for the quantitative analysis of mechanical parameters such as tissue velocity and tissue deformation [1-4]. TVI has been used clinically for many years in the field of cardiology, where the technique provides visual information on overall anatomy, regional movement- and velocity data of the myocardium together with quantitative measurements of these parameters (see [2,5,6] for an overview). All parameters are based on the velocity estimations derived from the phase shift in the ultrasound pulses that arises when they are reflected against a moving target. The method has been validated according to regional myocardial velocities and tested for inter- and intra-subject reproducibility [3,7-9]. Furthermore, TVI-based velocity and deformation parameters have been evaluated by our research group using several ultrasound scanners in a phantom study [10]. The parameters measured by TVI are considered to have a high clinical value in cardiology.

The research fields using TVI has broadened and several studies have reported using the technique on skeletal muscles [11-18]. However, there are likely important considerations to be made when applying this method, developed and evaluated for cardiac applications, in the musculoskeletal field. In cardiology the peak velocities are often of most interest. Myocardial peak velocities are normally in the range of 5–15 cm/s in resting conditions. In the studies of skeletal muscles the situation can be very different, for example in isometric contractions or contractions with low force level. For example, Peolsson et al. reported mean velocities of 0.08 cm/s in the trapezius muscle during shoulder elevations in myalgia patients [15].

In the standard ultrasound scanner settings the pulse repetition frequency (PRF) is set to a value in order to avoid aliasing artifacts when measuring myocardial velocities. Since the velocity range is divided into a fixed number of discrete values, we hypothesize that the standard settings may have a negative impact on the accuracy of very low velocity measurements, due to too large quantification steps. This study aims to evaluate TVI for estimation of the low tissue velocities found in low force level muscle contractions. An in-house developed phantom was used to see how different PRF-settings affected the accuracy of the velocity estimations.

## Methods

### The phantom

A phantom set-up, used in an earlier study evaluating tissue Doppler-based velocity and deformation parameters, was redesigned for the mimicking of skeletal muscle motion [10]. In this set-up a cylindrical tissue mimicking object was made of polyvinyl alcohol (PVA) (Sigma-Aldrich, St. Louis, Missouri, USA), with a length of 125 mm and a diameter of 40 mm. In order to get it sufficiently stiff 7 thaw cycles were used. Every thaw cycle constituted of a freezing period of 12 hours at a temperature of −18°C followed by 12 hours in room-temperature, resulting in 24 hour thaw cycles. In order to get speckles similar to that of muscle tissue a small amount of graphite powder (Merck, Darmstadt, Germany) was added to the PVA when mixed with water. The concentration was by mass; water (82%), PVA (15%) and graphite (3%). The speed of sound in tissue mimicking material was measured to lie within an interval of 1530–1580 m/s. The tissue mimicking object was immersed in a mixture of glycerol (Sigma-Aldrich, St. Louis, Missouri, USA) and deionized water. The concentration was by mass; deionized water (89%) and glycerol (11%). The chosen concentration resulted in a speed of sound of 1540 m/s. A single element transducer, an oscilloscope and a micrometer were used in the measurements to estimate the speed of sound in the tissue mimicking material and the immersing fluid. The difficulty in measuring the length of the soft PVA material with a micrometer in combination with the accuracy from reading the oscilloscope lead to the inaccuracy in the measurement of the tissue mimicking material. The fluid was placed in a large plastic container, which enabled the sound to travel a much longer distance than in the phantom material, and thus the better precision.

**Figure 1:**
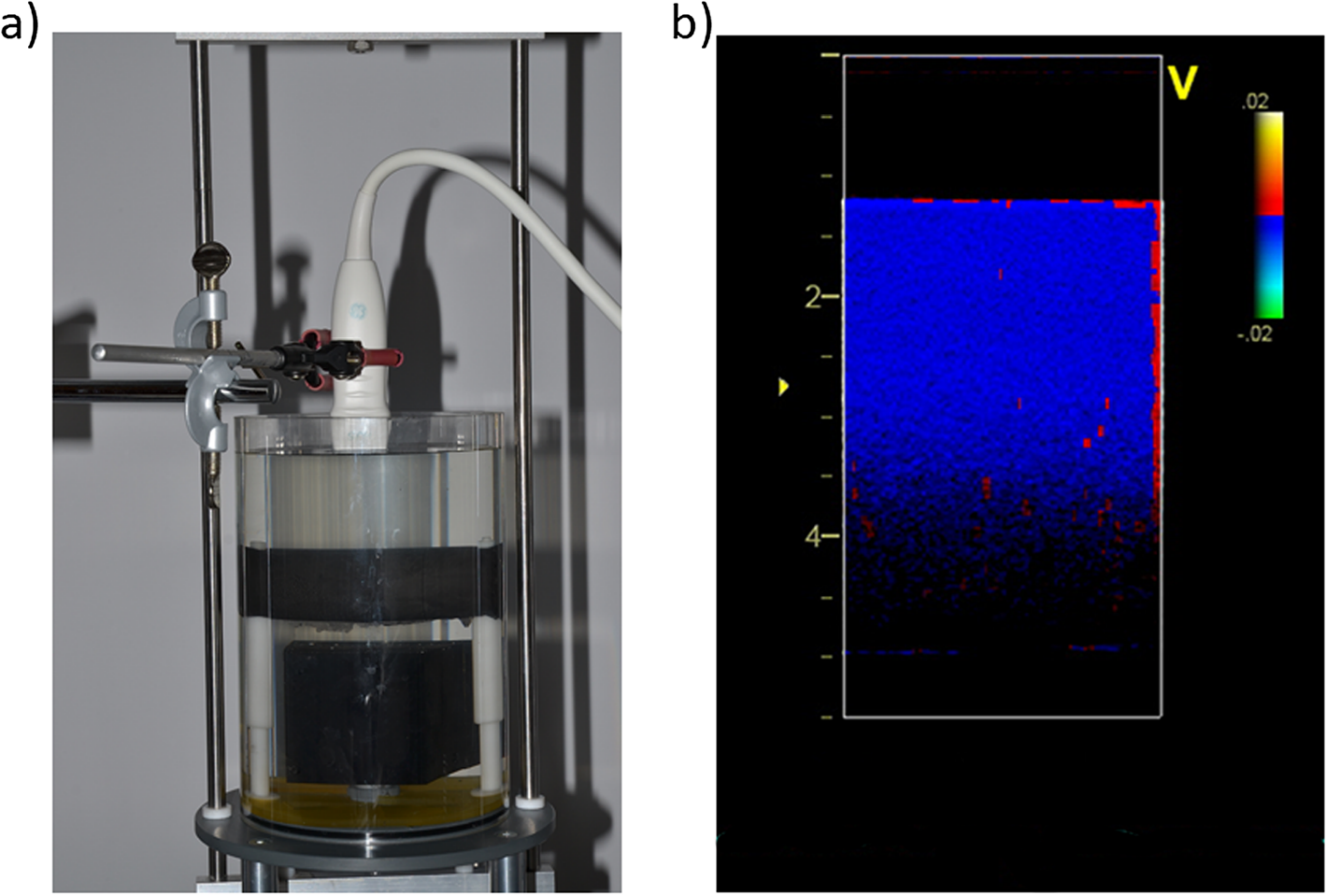
**The phantom setup. a**) shows the tissue mimicking material connected to the force generator and the linear transducer fixated above the water container. **b**) is an example of the resulting grayscale images from the ultrasound recordings. The velocity information from the TVI is color-coded and superimposed on the grayscale image. The blue color illustrates movement away from the ultrasound probe.

The force generator of the dynamic phantom was an ElectroPuls E3000 (Instron, Norwood, Massachusetts, USA), normally used for dynamic testing of material properties. The ElectroPuls E3000 can be programmed to perform motions of almost any wave form, and its performed motion is measured very accurately, making it possible to compare values measured by an ultrasound system with a true value. The tissue mimicking material was connected, in the distal parts, to the ElectroPuls E3000 by two plastic rods. In order to minimize the risk of reverberation artifacts a large rubber cube was place under the midsection coinciding with the transducer position. The acoustic properties of the rubber cube effectively absorb any entering ultrasound pulse (seen in Figure 1a).

The phantom was programmed to produce three different sine wave motions, with the frequency of 0.05 Hz and amplitudes of 1mm, 5mm and 8mm, resulting in mean peak velocities of 0.03 cm/s, 0.17 cm/s and 0.26 cm/s respectively (see Table 1).

**Table 1:** Phantom motion and ultrasound settings

**Phantom motion**	**Frequency**	**Amplitude**	**Mean peak velocities**	**Phantom repeatability – SD of displacement (mm)**
Sine wave	0.05 Hz	1 mm	0.03 cm/s	0.001
Sine wave	0.05 Hz	5 mm	0.17 cm/s	0.001
Sine wave	0.05 Hz	8 mm	0.26 cm/s	0.001
**Ultrasound settings**				
PRF (kHz)	Frame rate	(/s)		
0.25	66			
1.0	168			
1.75	214			

Listed in the table are the input parameters of the phantom motion together with the frame rates corresponding to the different PRF settings tested in the study.

**Figure 2:**
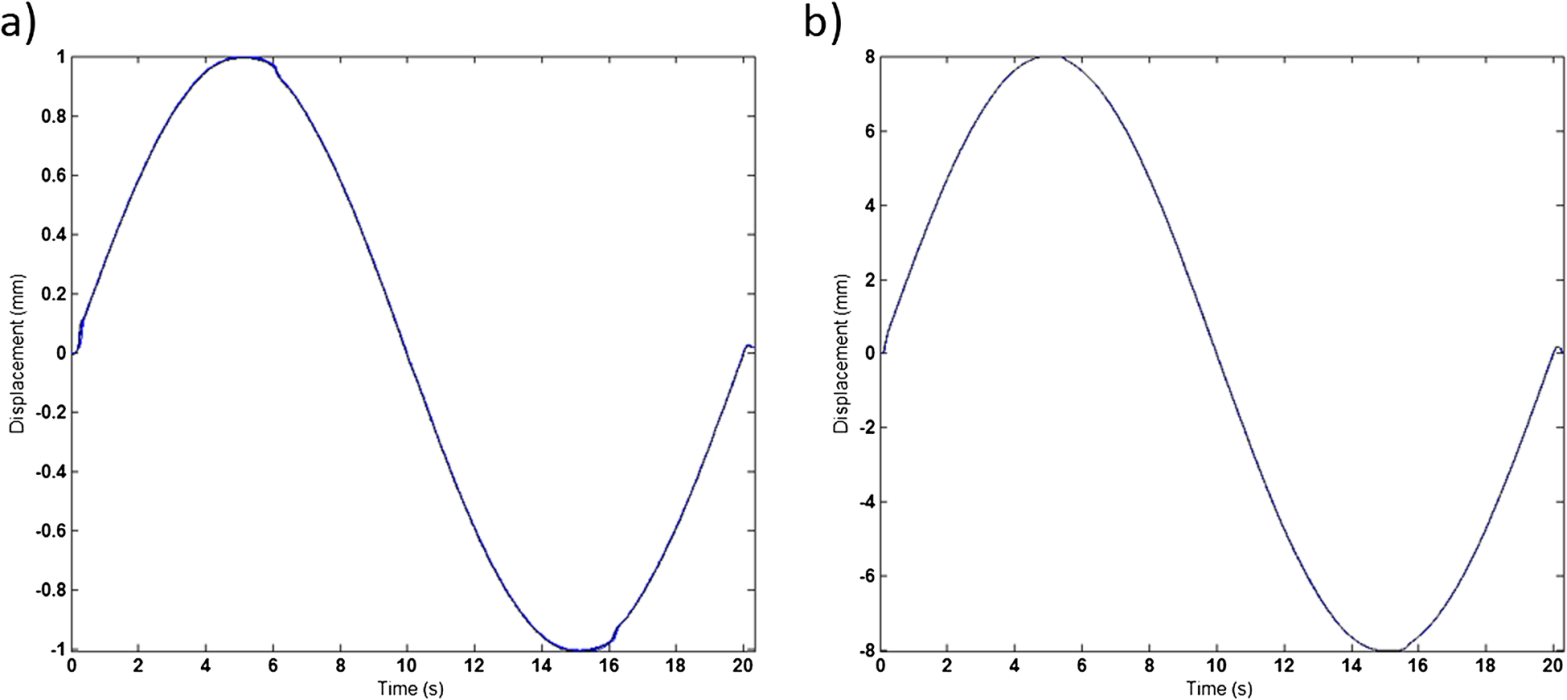
**Phantom repeatability.** Figure 2 shows two of the sine wave motions and the repeatability of the phantom, **a**) sine wave with 1 mm amplitude and **b**) sine wave with 8 mm amplitude. The 30 repetitions of each sine wave are displayed in the figure (10 repetition × 3 different PRF settings).

The motion performed by the electric motor of the ElectroPuls E3000 was registered by a built-in sensor on the motor shaft. The sensor measured the motor position relative to the starting position and the position was measured 1000 times per second. These position values were used to calculate the velocity of the performed motion. The repeatability of the phantom was evaluated based on the phantom data from the actual tests of the ultrasound systems, and the standard deviation was calculated to be ≤ 0.001 (mm) in all three displacement peaks, which were calculated separately in the three different sine wave motions. In Figure 2 the repeatability of the phantom for the sine waves with amplitude 1 mm and 8 mm is shown.

### Ultrasound scanner and settings

The movement of the tissue mimicking material was recorded with an ultrasound scanner (Vivid7, GE Vingmed, Horten, Norway) using a 12 MHz linear transducer. The acquisition depth and width was set to 5.5 cm and 2.5 cm respectively and the different PRF settings yielded TVI frame rates from 66 to 214 frames per second. One focus point was used at a depth of 2.5 cm. An example of a TVI color-coded grayscale image is shown in Figure 1b.

### Representation of velocity values

In the GE equipment the tissue velocities are estimated through autocorrelation of the phase shift of several consecutive returning ultrasound pulses. The information is translated into discrete values when the signal is converted from analog to digital and velocity is represented in a vector which is quantified by an equal number of positive and negative values, but not the value zero. The highest velocity value and the order of magnitude of each of the quantification steps depend on the chosen PRF setting. A lower PRF value will result in smaller steps in the velocity vector, thus providing a more accurate representation. However, limiting the PRF will increase the risk of aliasing artifacts since the highest value in the velocity vector also will lower. According to the Nyquist theorem the distance of the phase shift should be less than half a wavelength of the sent out pulses. Since the PRF sets the time delay between the ultrasound pulses it will therefore determine the highest velocity before aliasing occurs, also known as the Nyquist limit. Three different PRF settings were included in the test protocol; 0.25 kHz, 1.0 kHz and 1.75 kHz, where 1.0 kHz is the default setting.

### Test protocol and statistical analysis

The ultrasound probe was placed in the center of the tissue mimicking material at the phantom liquid surface. In the starting position the distance between the probe and tissue mimicking material was 1.2 cm. Ultrasound recordings were made while the phantom repeated one cycle of the sine motion. Ten repeated recordings were made with the three PRF settings on the three sine waves, yielding 90 recordings in total. The ultrasound probe was replaced before each recording.

The velocity information was extracted offline from the GE software EchoPac (version BT-08, GE VingMed, Horten, Norway) in a sample area of 8×16 mm centered at an image depth of 2.75 cm, in which the software calculates the mean velocity. The data was then further analyzed in MATLAB (2011b, Mathworks, Nattick, MA, USA). The phantom displacement values were used to calculate the velocity of the tissue mimicking material over time and then compared to the ultrasound velocity data. A median filter was implemented to reduce the noise in both signals.

The mean difference and standard deviation (SD) between performed and estimated velocity were calculated for the absolute peak values (three peaks) of the velocity curve. The percentage error of the true values was also calculated.

## Results

Table 2 presents the mean difference and SD between the peak velocity values from the phantom and estimated peak velocity values from EchoPac. The error is presented as the percentage of the true value.

**Table 2:** Mean difference and estimation error

**Velocity peak value (cm/s)**	**PRF (kHz)**	**Mean difference ± SD (cm/s)**	**Mean error (%)**
0.03	0.25	0.009 ± 0.006	31
	1.0	0.045 ± 0.006	149
	1.75	0.094 ± 0.007	313
0.17	0.25	0.034 ± 0.020	20
	1.0	0.032 ± 0.016	19
	1.75	0.029 ± 0.025	17
0.26	0.25	0.057 ± 0.036	22
	1.0	0.064 ± 0.039	24
	1.75	0.057 ± 0.026	22

Table 2 show the mean differences and the standard deviation of the peak velocity values for the three sine wave motions together with the mean error (presented as the percentage of the true value).

**Figure 3:**
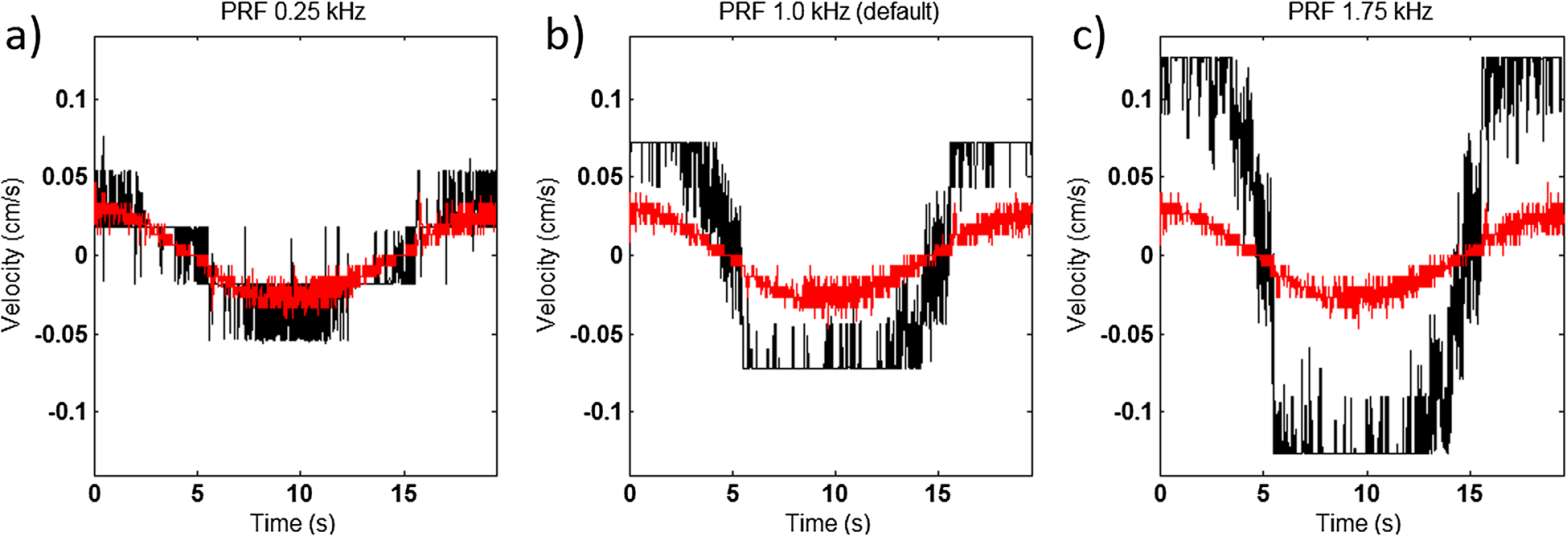
**Phantom velocity vs TVI estimated velocity 1.** The figure illustrates how the PRF setting affects the resolution of the TVI velocity estimation for phantom peak velocity at 0.03 cm/s, using the three different PRF settings **a**) 0.25 kHz **b**) 1.0 kHz **c**) 1.75 kHz. The red lines represent the phantom velocity and the black lines the TVI velocity of the unfiltered signals.

**Figure 4:**
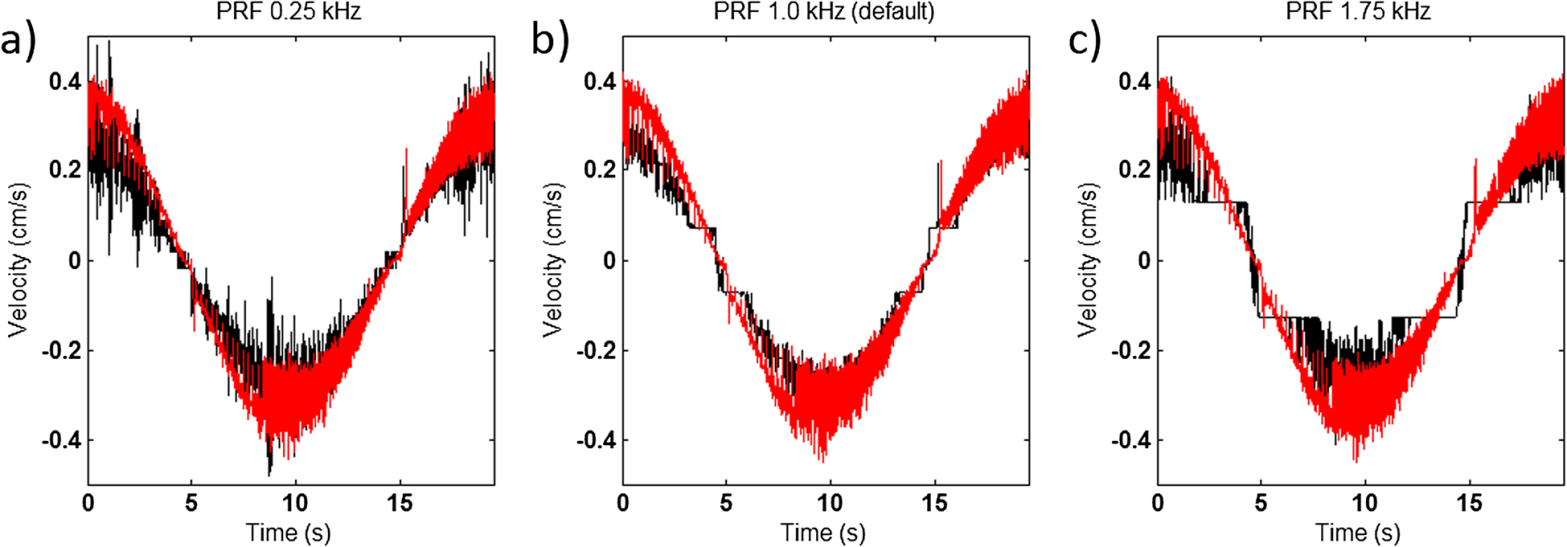
**Phantom velocity vs TVI estimated velocity 2.** The figure illustrates how the PRF setting affects the resolution of the TVI velocity estimation for phantom peak velocity at 0.26 cm/s, using the three different PRF settings **a**) 0.25 kHz **b**) 1.0 kHz **c**) 1.75 kHz. The red lines represent the phantom velocity and the black lines the TVI velocity of the unfiltered signals.

At very low velocities (< 0.03 cm/s) there are large differences in the TVI estimated velocities depending on the PRF-setting. With phantom peak velocity at 0.03 cm/s the error ranged from 31% up to 313% with the different PRF-settings in this study (see Figure 3). Furthermore, at the default PRF-setting (1.0 kHz) all the three peak values in all repeated measurements were estimated to 0.0724 cm/s or −0.0724 cm/s. This demonstrates the lowest possible quantification value at that setting and corresponds to an error of approx 160% of the true peak velocity. With peak velocities at 0.17 cm/s and 0.26 cm/s there is no difference in error with the three tested PFR settings, it is kept approximately around 20% (see Figure 4).

## Discussion

In this study TVI was evaluated for estimation of very low tissue velocities. An in-house developed phantom was used to see how different PRF-settings affected the accuracy of the velocity estimations. The results from the study show that for tissue velocities with peak velocities at 0.03 cm/s the PRF setting is crucial for the accuracy of the Doppler velocity estimation. However, at tissue peak velocities of order 0.3 cm/s the PRF setting has less effect on the error of the estimation.

In the analysis of the tissue mechanics, cardiac and musculoskeletal, both movement and deformation parameters are often used, such as displacement, velocity, strain, strain rate. All these parameters can be quantified and analyzed using TVI. However, it is the velocity parameter that is estimated using the autocorrelation method. The deformation parameters are calculated through the spatial gradient and temporal integration of the velocity information. This is done automatically in the off-line software. Thus, the inaccuracy of the velocity parameter will be transferred to the other parameters. If the parameter strain would have been analyzed instead it would have been more difficult to analyze how much of the error that directly could be connected to the PRF setting and how much that would be due to the calculating software, that includes filter functions etc. We chose to only evaluate the velocity parameter in this study and therefore a non-strained phantom was used to keep the motion as homogenous as possible. The performed motion was kept in one dimension (along the ultrasound beam) eliminating errors due to out of plane motion and possible angular errors. Furthermore, the transducer was completely fixed during the acquisitions to limit any error due to transducer movements. The sine wave motion was mainly chosen for the possibility to analyze both positive and negative peak velocity values and at the same time avoid rapid acceleration of the phantom motion.

The post-processing software which calculates the deformation parameters such as strain and strain rate has earlier been evaluated for cardiac applications by Mårtensson et al. They found considerably varying results in strain and strain rate between different manufactures and also between different workstations from the same manufacture [10]. In the same study two scanners of the same type used in this study were included, however using a phased array transducer during the acquisitions. One of those scanners was equipped with exactly the same software version as the scanner used in this study. The mean error found when using that scanner for peak velocities in the range of 8–9 cm/s was in the same order as found in this study. Combined, this suggests that there is reason to believe that one can expect a mean error of this magnitude when measuring TVI velocities in skeletal muscles. It should be pointed out that the difference between individual scanners and manufactures can be significant. In addition, Doppler strain rate has earlier also been evaluated in slowly moving tissue (0.01-0.1 cm/s) using a phantom mimicking the gastric wall. However in this study the PRF was kept very low through all measurements and instead the sample size and sample geometry for calculating strain rate were tested [19].

To avoid aliasing it is important to keep the Nyquist limit above the peak velocities. In the GE equipment this limit is displayed as the highest/lowest velocity value of the color coding-scale. Using the lowest possible PRF setting (0.25 kHz) in this study led to a Nyquist limit at 2.0 cm/s. Is that a sufficient peak limit for measurements of skeletal muscle tissue velocities? In general, the velocities will depend on the performed motion, type of contraction, produced force and maybe also what muscle. We also believe there is a large variation between individuals. A fast dynamic contraction can surely be in the same velocity range as the myocardium [20]. However, in studies of fatigue, muscle disorders and chronic pain conditions the performed task is more likely to be of either isometric or low force type and, as reported from Peolsson et al., the velocities may be much less than 1.0 cm/s [15].

Altogether, the clinical value of the accuracy and precision will be highly dependent on the measurement to be made and the intrinsic limitations of the used equipment. We believe that TVI can be a powerful tool when it comes to analysis of intramuscular mechanics with both high spatial and temporal resolution. It also becomes a complementary method to electromyography as it provides the possibility to analyze deeper located muscles non-invasively.

## Conclusions

Applying TVI on skeletal muscles one must be aware of the limitations that comes with the system. The results from the present study showed that the PRF setting did not seem to affect the accuracy of the velocity estimation at tissue velocities above 0.17 cm/s. However at lower velocities (0.03 cm/s) the setting was crucial for the accuracy. The PRF should therefore preferable be reduced when the method is applied in low-level muscle contraction. Further, the results indicate that there is an intrinsic error of the used scanner of approximately 20%. It should be carefully considered before the method is applied in a clinical setting if such an error is acceptable.

## Competing interests

LÅB is co-developer and former patent holder of the analyzing software package and is having ongoing research collaboration with GE. However, no financial support for the present study has been provided from GE. The other authors declare that they have no competing interests.

## Authors’ contributions

FL designed and coordinated this study. FL and MM performed th data collection, data analysis and drafted the manuscript. CG and LÅB interpreted the data and critically revised the manuscript. All authors read and approved the final manuscript.

## Pre-publication history

The pre-publication history for this paper can be accessed here:

http://www.biomedcentral.com/1471-2342/13/16/prepub
